# Pet ownership, dog types and attachment to pets in 9–10 year old children in Liverpool, UK

**DOI:** 10.1186/1746-6148-9-102

**Published:** 2013-05-13

**Authors:** Carri Westgarth, Lynne M Boddy, Gareth Stratton, Alexander J German, Rosalind M Gaskell, Karen P Coyne, Peter Bundred, Sandra McCune, Susan Dawson

**Affiliations:** 1 Institute of Infection and Global Health, Faculty of Health and Life Sciences, University of Liverpool, Leahurst Campus, Neston, Cheshire CH64 7TE, UK; 2 Physical Activity, Exercise and Health Research Group, Research Institute for Sport and Exercise Sciences, Liverpool John Moores University, Liverpool L3 2ET, UK; 3 Sport and Health Portfolio, College of Engineering Swansea University, 942c Talbot Building, Singleton Park, Swansea SA2 8PP, UK; 4 Institute of Ageing & Chronic Disease, Faculty of Health and Life Sciences, University of Liverpool, Leahurst Campus, Neston, Cheshire CH64 7TE, UK; 5 Institute of Psychology Health and Society, Faculty of Health and Life Sciences, University of Liverpool, Whelan Building, Quadrangle, Brownlow Hill, Liverpool L69 3GB, UK; 6 WALTHAM® Centre for Pet Nutrition, Waltham-on-the-Wolds, Melton Mowbray, Leics LE14 4RT, UK; 7 School of Veterinary Science, Faculty of Health and Life Sciences, University of Liverpool, Leahurst Campus, Neston, Cheshire CH64 7TE, UK

## Abstract

**Background:**

Little is known about ethnic, cultural and socioeconomic differences in childhood ownership and attitudes to pets. The objective of this study was to describe the factors associated with living with different pet types, as well as factors that may influence the intensity of relationship or ‘attachment’ that children have to their pet. Data were collected using a survey of 1021 9–10 year old primary school children in a deprived area of the city of Liverpool, UK.

**Results:**

Dogs were the most common pet owned, most common ‘favourite’ pet, and species most attached to. Twenty-seven percent of dog-owning children (10% of all children surveyed) reported living with a ‘Bull Breed’ dog (which includes Pit Bulls and Staffordshire Bull Terriers), and the most popular dog breed owned was the Staffordshire Bull Terrier. Multivariable regression modelling identified a number of variables associated with ownership of different pets and the strength of attachment to the child’s favourite pet. Girls were more likely to own most pet types, but were no more or less attached to their favourite pet than boys. Children of white ethnicity were more likely to own dogs, rodents and ‘other’ pets but were no more or less attached to their pets than children of non-white ethnicity. Single and youngest children were no more or less likely to own pets than those with younger brothers and sisters, but they showed greater attachment to their pets. Children that owned dogs lived in more deprived areas than those without dogs, and deprivation increased with number of dogs owned. ‘Pit Bull or cross’ and ‘Bull Breed’ dogs were more likely to be found in more deprived areas than other dog types. Non-whites were also more likely to report owning a ‘Pit Bull or cross’ than Whites.

**Conclusions:**

Gender, ethnicity and socioeconomic status were associated with pet ownership, and sibling status with level of attachment to the pet. These are important to consider when conducting research into the health benefits and risks of the common childhood phenomenon of growing up with pets.

## Background

The study of companion animal ownership and the physical, social and psychological health of people is an expanding research field, encompassed by the term ‘Human-Animal Interaction’ (HAI). Pets are proposed to confer both physiological and psychological health benefits [1-7], but the evidence is inconclusive [8]. There are also potential health risks associated with pet ownership including aggression and bites, allergies and zoonosis [1,9].

Much HAI research has focused primarily on pet owners with significant health challenges, rather than pet ownership by average people in everyday life [6]. It has also mainly considered adults; less is known about the role pets play in the lives and wellbeing of children, much of which is observational studies of child/pet interaction, or interviews with children about their attitudes and beliefs regarding animals [10]. In particular, a paucity of research into ethnic, cultural and socioeconomic differences in pet ownership and attitudes to pets has been noted [10]. The few studies that have focused upon the variation in HAI by ethnicity, have mainly been limited to black versus white responses in older age groups as opposed to young children [11-13]. Childhood experience of pets may vary between ethnic and cultural groups with differing attitudes towards animals, and this may influence individual behaviour and future decisions regarding animal ownership. Thus, experiences regarding pets during childhood have implications across the life course.

It is important to understand the factors associated with childhood pet ownership in order to evaluate the benefits and risks involved in such ownership. A number of demographic variables such as age, gender, socioeconomics and ethnic status are known to be associated with many human health behaviours [14], and also types of pet ownership in adults [15-18]. A recent UK study, using a well-characterised longitudinal birth cohort, provided data on a number of factors associated with pet ownership during childhood up to age 10 years, including socioeconomic variables, ownership of other pet types, and a parental history of pet ownership [19]. However, this study lacked information about the relationship or interactions of the children with their pets, or detail about the pet breed or type.

It has been suggested that HAI data collection should be incorporated into on-going or new studies planned on other topics, as a cost-effective method to gather important cross-sectional information about pets in the home and impacts on aspects of child health and development [10]. To this end, the current study used an established programme of child health and fitness monitoring to access and sample a cross-section of 9–10 year old primary school children in Liverpool, UK, [20,21] about their ownership and interactions with pets, and to link this to demographic information. The study aimed to describe pet ownership and contact with animals owned by others, in 9–10 year old children attending primary schools in a deprived area of a UK city. It also aimed to investigate the factors that may be associated with ownership of different pet types, including certain types of dogs (Pit Bulls and other ‘Bull Breeds’), as well as factors that may influence the intensity of relationship or ‘attachment’ that children have to their pet.

## Methods

### Data collection

Annually, all primary schools within Liverpool Local Education Authority are invited to participate in the *Sports*Linx project [20,21]. From the participating primary schools, all 9–10- year-old children are invited to take part. Participants complete a range of field-based fitness assessments during a Fitness Fun Day conducted at local leisure centres by experienced Liverpool City Council Fitness Officers. Participation is subsequent to granted informed parental consent and participant assent, and after the completion of medical screening forms. Ethical approval for the addition of the Child Lifestyle and Pets (CLAP) questionnaire to a sample of the 2010–2011 *Sports*Linx data collection was obtained from the North West 3 Research Ethics Committee – Liverpool East.

One thousand and ninety one 9–10 year-old children were sampled over ten weekdays in Oct-Nov 2010. They attended 31 schools present at the *Sports*Linx Fitness Fun Days in Wavertree, Liverpool. Children were asked to complete the questionnaire for the household that they spent most of their time in. This was conducted at one ‘station’ on the rotation of exercises that they were performing as part of their fitness testing. For each session, two University of Liverpool employees (one being the first author) supervised data collection, all of whom were trained in assisting children to complete the questionnaire. When available, schoolteachers and other *Sports*Linx instructors also assisted.

The full questionnaire can be requested from the authors. Questions asked concerned: pet types currently owned (including length of time owned and type of dog); for those that did not currently own a pet, the pets owned in the last 5 years; and contact with other animals outside their main home. The strength of the relationship between the child and their ‘favourite’ pet currently owned (often termed ‘attachment’) was measured using a validated series of 27 questions with a Likert-type scale; the CENSHARE Pet Attachment Scale [22]. Demographic information about the household and environment was also collected, including: number of siblings living in the household; and whether or not the child was the youngest. Parental consent forms were used to collect other data on gender, ethnicity (White UK, Black UK, African, Indian, Pakistani, Bangladeshi, Chinese, Somali, mixed or ‘other ethnicity’) and home postcode.

### Data analysis

Ethnicity data were further categorised into white or non-white for statistical purposes. Children were categorised as being a single child (no brothers or sisters), the youngest child, or neither single nor the youngest. Index of Multiple Deprivation 2007 (IMD2007) score was calculated from home postcode. An overall attachment score was determined by summing the individual question responses into a total CENSHARE Score after reverse scoring items 2, 13, 19, 20 and 27.

Data were initially analysed in MINITAB using chi-squared tests and binary or nominal logistic regression for categorical variables. The continuous variable of CENSHARE (attachment) score was analysed using T-tests or Analysis of Variance, and regression, as it was normally distributed. The continuous variable of IMD2007 score was analysed using the Kruskal-Wallis test as it was not normally distributed. Adjusted models were built for the outcomes pet type, dog type and attachment score using multivariable modelling of fixed effects: gender; ethnicity; sibling status; deprivation score; and, for the outcome of attachment, pet type. Models were also tested in MLwiN with the additional hierarchical structure of ‘Day of recording’ and ‘School’ as random effects; there were negligible differences in the estimates and so standard models are presented here.

## Results

### Response

One thousand and twenty four children completed CLAP questionnaires, and this represented 94% of those attending. Reasons for non-completion were lack of time to survey all the children or very occasionally lack of capability of the child to complete the questionnaire due to non-English speaking or learning difficulties. Three questionnaires were discarded at the data entry stage for looking non-reliable; thus, CLAP questionnaire data were available for 1021 children (94%). Some schools opted to keep the consent forms on their premises and, therefore, complete demographic data were available for 88.6% of the total intended sample (n=967).

### Sample demographics

Two hundred and eighty-nine children (32.2%) reported that they also spent time living somewhere else as well as the main home they were answering the questions for. Nine hundred and seventy-nine (96.9%) children lived in a house, as opposed to a flat or other type of accommodation. One hundred and eighteen children (11.7%) were single children and 374 (42.8%) children stated that they were the youngest of their siblings. Five hundred and thirteen (53%) children were male. Six hundred and eighty three (84.1%) were white (UK) ethnicity and 17 (2.1%) Black (UK), others being of African (8), Indian (9), Pakistani (6), Bangladeshi (6), Chinese (8), Somali (10), mixed or other ethnicity (69). IMD2007 was reported for 796 children and score had a median of 40.85, range 6.5-81.3.

### Pet ownership and time pet owned

Six hundred and eighty (66.8%) children reported owning a pet, only 3 children did not provide this information (see Table 1). 37.1% reported owning a dog(s), ranging from one (75.5%) to nine (2 children); 16.6% a cat, ranging from one (64.2%) to seven (1 child). There was evidence of a difference between length of time owned and pet type (Chi-squared P<0.001) likely reflecting the differing longevity of pet types.

**Table 1:** Prevalence of pet ownership in 9-10year old children in Liverpool, n=1018

		**Time pet type owned (P<0.001)**			
**Pet type**	**Yes, n (%)**	**<1yr**	**1-5yrs**	**>5yrs**	**All my life**
Any pet	680 (66.8)				
Dog	378 (37.1)	59 (17.2)	132 (38.4)	70 (20.4)	83 (24.1)
Cat	169 (16.6)	23 (15.3)	48 (32.0)	28 (18.7)	51 (34.0)
Rabbit	93 (9.1)	24 (27.6)	43 (49.4)	12 (13.8)	8 (9.2)
Rodent	149 (14.6)	51 (38.1)	57 (42.5)	15 (11.2)	11 (8.2)
Horse	21 (2.1)	4 (20.0)	6 (30.0)	7 (35.0)	3 (15.0)
Other pet	367 (36.1)	81 (24.7)	125 (38.1)	55 (16.8)	67 (20.4)

### Factors associated with pet ownership

Factors associated with ownership of pets in general, dogs, and cats, after adjustment, are shown in Table 2. Full data are not shown for other pet types. Females were more likely than males to report owning all pet types except rabbits (Dog OR=1.47, 95%CI=1.08-2.01, P=0.02; Cat OR=1.58, 95%CI=1.08-2.32, P=0.02; rodent (marginal) OR=1.49, 95%CI=0.99-2.24 P=0.06; horse OR=12.94, 95%CI=1.66-101.05, P=0.02; other pet OR=1.41, 95%CI=1.04-1.91, P=0.03). Compared with white children, non-whites were less likely to own dogs (OR=0.23, 95%CI=0.13-0.39, P<0.001), rodents (OR=0.29, 95%CI=0.13-0.66, P=0.003) or other pets (OR=0.35, 95%CI=0.21-0.57, P<0.001), but there was no evidence of a difference for cats, rabbits or horses. Interestingly, no Indian or Pakistani/Bangladeshi children reported dog ownership. Due to low numbers in ethnic sub-groups, univariable and adjusted models could not be reported at this level.

**Table 2:** Multilevel multivariable models of factors associated with pet ownership, dog ownership and cat ownership

**Outcome (predictor variable)**	**No (n or median)**	**Yes (n or median)**	**Crude OR**	**Crude 95%CI**	**Crude P**	**Adjusted OR**	**Adjusted 95% CI**	**Adjusted P**
**a) Any pet**								
Gender								
Male	199	312	1					
Female	117	336	1.83	1.39-2.41	<0.001	2.00	1.44-2.79	**<0.001**
Ethnicity								
White	174	507	1					
Non-white	76	53	0.24	0.16-0.35	<0.001	0.23	0.15-0.35	**<0.001**
Sibling status								
Neither	183	311	1					
Single	37	80	1.27	0.83-1.96	0.27	1.00	0.59-1.68	1.00
Youngest	110	264	1.41	1.06-1.88	0.02	1.17	0.82-1.67	0.38
Deprivation score	39.2	41.2	1.00	0.99-1.01	0.86	1.01	1.00-1.02	0.06
**b) Dog**								
Gender								
Male	338	173	1					
Female	270	183	1.32	1.02-1.72	0.04	1.47	1.08-2.01	**0.02**
Ethnicity								
White	397	284	1					
Non-white	106	23	0.30	0.19-0.49	<0.001	0.23	0.13-0.39	**<0.001**
Sibling status								
Neither	322	172	1					
Single	79	38	0.90	0.59-1.38	0.63	0.79	0.46-1.33	0.37
Youngest	220	154	1.31	0.99-1.73	0.06	1.17	0.84-1.63	0.35
Deprivation score	33.1	48.7	1.02	1.01-1.02	<0.001	1.02	1.01-1.03	**<0.001**
**a) Cat**								
Gender								
Male	444	67	1					
Female	356	97	1.81	1.28-2.54	0.001	1.58	1.08-2.32	**0.02**
Ethnicity								
White	552	129	1					
Non-white	114	15	0.56	0.32-1.00	0.05	0.69	0.38-1.26	0.23
Sibling status								
Neither	419	75	1					
Single	94	22	1.29	0.77-2.19	0.34	0.98	0.52-1.85	0.94
Youngest	308	66	1.20	0.83-1.72	0.33	1.13	0.75-1.70	0.57
Deprivation score	40.85	40.85	1.00	0.99-1.01	0.87	1.00	0.99-1.01	0.98

a) n=742. Hosmer-lemeshow=0.42.

b) n=742. Hosmer-lemeshow=0.53.

a) n=742. Hosmer-lemeshow=0.85.

There was no evidence that having a pet was associated with the number of siblings a child had or being a single child. On univariable analysis, children who were the youngest appeared to be more likely to own pets, but this association disappeared after adjustment (Table 2). There was no evidence that whether a pet was owned varied by deprivation score (IMD2007) for any pet type other than dogs (OR=1.02, 95%CI=1.01-1.03, P<0.001, Table 2). Comparing scores, those without dogs (n=501) scored a median of 33.1, compared to median 48.7 for those with dogs (n=293, P<0.001; a higher score equates to more deprivation). There also appeared to be a dose–response effect, with children with multiple dogs having even higher deprivation scores than those with just one dog, medians 46.3 (n=219) and 57.7 (n=71) respectively.

### Previous pets owned

Children who did not currently own a pet were asked to indicate whether they had owned a pet in the last five years, and 168 (52.2%) indicated that they had. Fifty seven indicated (17.8%) owning a dog, 28 (8.7%) a cat, 21 (6.6%) a rabbit, 30 (9.4%) a rodent, none owning a horse, and 93 (29.1%) an ‘other’ pet.

### Dog types

The dog-owning children indicated owning a total of 536 dogs; two children indicated owning a dog but did not indicate how many. Individual data were collected on 505 dogs (as children were asked to describe up to 3 dogs). For 65 dogs, no type indication was given. The most common breed was the Staffordshire Bull Terrier (n=75, 18.9%) followed by Shih Tzu (n=38, 9.6%). Overall, 43 dogs were indicated as mixed-breed (9.8%); the others were categorised as UK Kennel Club Groupings ‘Terrier’ (n=118, 26.9%; modified to include Jack Russell and Patterdale), ‘Utility’ (n=80, 18.2%), ‘Toy’ (n=60, 13.7%), ‘Gundog’ (n=57, 13.0%), ‘Working’ (n=32, 7.3%), ‘Other’ (n=23, 5.2%; including Pit Bull, American Bulldog, American Bullmastiff and Presa Canario), ‘Pastoral’ (n=19, 4.3%), and ‘Hound’ (n=7, 1.6%). It must be noted that simply ‘Bulldog’ was categorised as Utility due to the definition for UK Kennel Club Utility Group, although it is likely that some or all of these may have actually been the ‘American Bulldog’ rather than the English or British Bulldog as assumed; the American version is anecdotally popular in this area. Seventeen dogs were reported to be a pure ‘Pit Bull’ or pseudonym for Pit Bull e.g. ‘Red Nose’, ‘Irish Staff’ or ‘Irish Blue’ [23].

Of the children who reported owning at least one dog, 22 (5.8%) reported that they lived with a Pit Bull or stated Pit Bull cross; this equated to 2.2% of all children sampled living with a stated ‘Pit Bull or cross’. If this categorisation were to be extended to all breeds and crosses of Bull Breeds, such as Pit Bulls, Staffordshire Bull Terrier, or Bulldog (often the American Bulldog) then 27.3% (n=103) of children who owned dogs had a ‘Bull Breed’ dog, or 10.1% of the total children sampled were living with one. Children who owned a ‘Pit Bull or cross’ were from more deprived areas than those with other types of dog (Kruskal-Wallis P<0.001, median 66.4 (n=16) compared to 45.0 (n=277); Table 3, OR=1.06, 95%CI=1.02-1.11, P=0.01). Similarly, those with a ‘Bull Breed’ dog were also from more deprived areas than children with other dog type (Kruskal-Wallis P<0.001, median 55.1 (n=81) compared to 41.55 (n=211); Table 3, OR=1.03, 95%CI=1.01-1.05, P<0.001). There was also some evidence that non-white ethnicities were four times more likely to report owning a ‘Pit Bull or cross’ (OR=3.92, 95%CI=0.99-15.59, P=0.05) than white children, but not the broader category of Bull Breed (Table 3). There was no evidence that dog type varied by gender or sibling status. The Hosmer-Lemeshow statistics (Table 3) were low indicating that the models did not fit particularly well, suggesting that other influences existed on dog type than those measured here.

**Table 3:** Factors associated with ownership of a ‘Pit Bull or cross’ or a ‘Bull Breed’ dog

**Outcome (predictor variable)**	**No (n or median)**	**Yes (n or median)**	**Crude OR**	**Crude 95%CI**	**Crude P**	**Adjusted OR**	**Adjusted 95% CI**	**Adjusted P**
**a) Pit Bull or cross**								
Gender								
Male	160	13	1					
Female	176	7	0.49	0.19-1.26	0.14	0.49	0.15-1.59	0.23
Ethnicity								
White	269	15	1					
Non-white	19	4	3.78	1.14-12.50	0.03	3.96	0.99-15.76	**0.05**
Sibling status								
Neither	162	10	1					
Single	37	1	0.44	0.05-3.53	0.44	0.79	0.08-7.46	0.84
Youngest	146	8	0.89	0.34-2.31	0.81	0.93	0.29-3.01	0.90
Deprivation score	46.98	66.40	1.07	1.03-1.11	0.001	1.06	1.02-1.11	**0.01**
**b) Bull Breed**								
Gender								
Male	124	48	1					
Female	134	49	0.94	0.59-1.51	0.81	1.04	0.60-1.82	0.88
Ethnicity								
White	206	77	1					
Non-white	14	9	1.72	0.72-4.41	0.23	0.99	0.35-2.81	0.99
Sibling status								
Neither	119	52	1					
Single	31	7	0.52	0.21-1.25	0.14	0.62	0.22-1.70	0.35
Youngest	115	39	0.78	0.48-1.26	0.31	0.59	0.33-1.06	0.08
Deprivation score	41.55	55.05	1.03	1.02-1.05	<0.001	1.03	1.01-1.05	**<0.001**

a) n=274. Hosmer-lemeshow=0.20.

b) n=274. Hosmer-lemeshow=0.13.

Bull Breed defined as for example Pit Bull or Staffordshire Bull Terrier or American Bulldog, including crosses of these.

The dogs ranged in age from 0–20 years, mean 4.0, median 3.0 years, and the age of dog was right-skewed in its distribution. Sixty-nine (15.6%) of the dogs reportedly lived mainly outside. Nineteen (4.1%) of the dogs were reported to never be walked, 78 (16.9%) less than once a week; 170 (36.8%) several times a week, and 195 (42.2%) once a day or more.

### Attachment to pets

Six hundred and one children (88.4% of those who had pets) indicated their ‘favourite’ pet owned: 90 (15.0%) indicated a cat, 311 (51.8%) dog, 12 (2.0%) horse, 29 (4.8%) rabbit, 58 (9.7%) rodent, 74 (12.3%) fish and 27 (4.5%) ‘other’ pet (e.g. birds, lizards, tortoises and turtles, snakes, frogs). Thus, compared to the proportions of pet types owned, dogs were more likely to be indicated as the favourite pet owned, although they may have been the only pet. The mean CENSHARE attachment score was 55 (median 53, range 27–102), for those with complete attachment scales (n=381). Internal consistency was high (Cronbach’s alpha=0.90). Individual findings for each of the CENSHARE questions are displayed in Table 4.

**Table 4:** CENSHARE attachment scale answers for favourite pet reported as owned by child

**CENSHARE question, n (%)**	**Almost always**	**Often**	**Sometimes**	**Almost never**	**Missing**
1. Within your family, your pet likes you best	330 (57.0)	121 (20.9)	110 (19.0)	18 (3.1)	22
2. You are too busy to spend time with your pet	61 (10.7)	69 (12.1)	187 (32.8)	254 (44.5)	30
3. You spend time each day playing with or exercising your pet	200 (34.8)	154 (26.8)	120 (20.9)	100 (17.4)	27
4. Your pet comes to greet you when you arrive	343 (60.5)	67 (11.8)	71 (12.5)	86 (15.2)	34
5. You talk to your pet as a friend	263 (46.9)	90 (16.0)	111 (19.8)	97 (17.3)	40
6. Your pet is aware of your different moods	202 (35.9)	113 (21.1)	115 (20.5)	132 (23.5)	39
7. Your pet pays attention and obeys you quickly	205 (36.7)	102 (18.3)	138 (24.7)	114 (20.4)	42
8. You confide in your pet	209 (37.9)	85 (15.4)	96 (17.4)	161 (29.2)	50
9. You play with your pet when he/she approaches	324 (57.2)	102 (18.3)	77 (13.8)	54 (9.7)	44
10. You spend time each day training your pet	150 (25.6)	110 (20.1)	149 (27.2)	149 (27.2)	53
11. You show photos of your pet to your friends	165 (30.3)	82 (15.1)	112 (20.6)	186 (34.1)	56
12. You spend time each day grooming your pet	105 (18.9)	115 (20.7)	149 (26.8)	187 (33.6)	45
13. You ignore your pet when he/she approaches	50 (9.2)	20 (3.7)	53 (9.7)	421 (77.4)	57
14. When you come home, your pet is the first one you greet	297 (54.8)	69 (12.7)	88 (16.2)	88 (16.2)	59
15. Your pet tries to stay near you by following you	247 (45.4)	88 (16.2)	107 (19.7)	102 (18.8)	57
16. You buy presents for your pet	212 (39.0)	95 (17.5)	152 (28.0)	84 (15.5)	58
17. When you feel bad, you seek your pet for comfort	275 (50.6)	87 (16.0)	86 (15.8)	96 (17.7)	57
18. You prefer to be with your pet more than with most people you know	161 (29.7)	107 (19.7)	172 (31.7)	103 (19.0)	58
19. When your pet misbehaves you hit him/her	35 (6.5)	25 (4.6)	55 (10.2)	426 (78.7)	60
20. Your pet is a nuisance and a bother to you	65 (12.2)	39 (7.3)	84 (15.8)	345 (64.7)	68
21. You consider your pet to be a member of the family	431 (80.0)	33 (6.1)	36 (6.7)	39 (7.2)	62
22. When you feel bad, you seek your pet for comfort	281 (52.5)	74 (13.8)	95 (17.8)	85 (15.9)	66
23. You feel sad when you are separated from your pet	222 (41.4)	78 (14.6)	126 (23.5)	110 (20.5)	65
24. You like to have your pet sleep near your bed	218 (41.3)	40 (7.6)	87 (16.5)	183 (34.7)	73
25. You like to have your pet sleep on your bed	194 (36.7)	39 (7.4)	54 (10.2)	241 (45.6)	73
26. You like to have your pet near you when you study, read or watch TV	255 (47.7)	57 (10.7)	102 (19.1)	121 (22.6)	66
27. You don’t like your pet to get too close to you	54 (10.3)	27 (5.2)	45 (8.6)	396 (75.9)	79

CENSHARE total score varied by type of favourite pet indicated (ANOVA P<0.001) with the least scores (most attached) being for dogs (mean score 49) and greatest scores (least attachment) being for fish (mean score 79), as would be expected from the nature of the questions. The adjusted model of attachment (Table 5) suggested that compared to cats, children scored a greater attachment to dogs (mean difference −6.45 points, P=0.002) and a lesser attachment to fish (+25.64, P<0.001) and ‘other’ pets (+16.88, P=0.003). Thus, it is important that analysis of attachment score accounts for pet type either by adjustment or stratification. There was good evidence that single children (no brothers or sisters) reported stronger attachment to the favourite pet than those who were neither a single child nor the youngest child (−9.54, P<0.001). There was also some evidence that the youngest child also reported stronger attachment (−3.16, P=0.05). There was no evidence that gender, ethnicity or deprivation score was associated with attachment score (Table 5). When dogs were analysed separately, there was no evidence that dog type (Pit Bull or Bull Breed) was associated with attachment score (data not shown).

**Table 5:** Factors associated with attachment to favourite pet (CENSHARE Pet Attachment Scale total score; lower=more attached)

**Outcome (predictor variable)**	**Mean score**	**Crude Coef**	**Crude SECoef**	**Crude P**	Adjusted* Coef	Adjusted* SECoef	**Adjusted P**
**Attachment score**							
Pet type							
Cat	56.28						
Dog	49.09	−7.21	1.94	<0.001	−6.45	2.09	**0.002**
Horse	52.13	−4.15	5.02	0.41	−10.90	7.75	0.16
Rabbit	54.53	−1.75	3.51	0.62	−2.45	3.91	0.53
Rodent	56.62	0.34	2.74	0.90	1.19	3.00	0.69
Fish	79.46	23.18	2.83	<0.001	25.64	3.03	**<0.001**
Other	72.07	15.79	3.96	<0.001	16.88	5.66	**0.003**
Gender							
Male	54.7						
Female	55.0	0.34	1.70	0.84	0.13	1.53	0.93
Ethnicity							
White	54.5						
Non-white	55.6	1.10	3.13	0.73	−0.47	2.72	0.86
Sibling status							
Neither	58.14						
Single	50.43	−7.71	2.60	0.003	−9.44	2.57	**<0.001**
Youngest	52.46	−5.68	1.76	0.001	−3.16	1.62	**0.05**
Deprivation score	-	−0.06	0.04	0.18	0.03	0.04	0.49

*n=298.

### Contact with other animals

All children were also asked to indicate whether they had contact with animals outside their home, for example belonging to a family member or friend (i.e. not their own pet); 72.2% (n=697) indicated yes, and reported the frequency (daily, weekly or monthly) that they contact that animal type (Table 6). Dogs were the animal most frequently contacted.

**Table 6:** Child indication of animals other than their own pet contacted (n=965), and frequency of contact

**Animal**	**Yes, n (%)**	**Daily, n (%)**	**Weekly, n (%)**	**Monthly, n (%)**
Dog	533 (55.6)	199 (38.5)	185 (35.8)	133 (25.7)
Cat	328 (34.2)	121 (40.3)	103 (34.3)	76 (25.3)
Rabbit	131 (13.7)	42 (34.2)	41 (33.3)	40 (32.5)
Rodent	99 (10.3)	39 (42.4)	21 (22.8)	32 (34.8)
Horse (including riding)	73 (7.6)	19 (27.5)	22 (31.8)	28 (40.6)
Other pet	190 (19.8)	62 (35.8)	57 (33.0)	54 (31.2)

## Discussion

This study provides much novel and confirmatory information on sociodemographic factors associated with childhood ownership of different pet types, including those that may influence the nature of the relationship children have with their pets. Such data are likely to be useful to researchers in the fields of public health, social science and veterinary science, as well as those studying the field of human-animal interactions. We have demonstrated that children are keen to tell us all about their pets, regardless of pet type, and we suggest that in order to maximise compliance and understanding of the study, researchers should be present during data collection with the children where possible.

A strength of this study is that the sample of children was relatively large, not convenience-based, and had high response rates, due to the specific context of data collection where all children were captured for a time period set aside purely for this purpose. We also used multivariable regression modelling, to adjust for the confounding effects that demographic variables can have on each other, which are methods lacking from much previous HAI literature.

However, the data are limited by the nature of self-report, as we did not see the actual pets or the dog types ourselves for verification, nor did we ask the parents. The nature of what constitutes pet ‘ownership’ may also be questioned, and may differ between adults and children, but for the purposes of this study, both the children and investigators inferred it to mean living with a pet in the household in which they spent most of their time, or in the case of horses, the child feeling that the horse belonged to their household. We also did not ask whether the children were from dual or single-parent families, which may have been interesting in terms of attachment to pets. The data were collected from a specific population, 9–10 year-old children attending primary schools in a region of Liverpool that contains areas of considerable deprivation and, thus, may not be generalisable to other UK cities or countrywide, or other age groups. In the 2001 Census the Liverpool Local Authority Area was noted to have lower employment, fewer people with qualifications and lower rates of home ownership, when compared with the whole of England [24]. The study was also cross-sectional and, therefore, causation cannot necessarily be implied.

Dogs are often thought to be the most common pet type owned by households in the UK, closely followed by cats [15,16,25]. However, in this study, dogs were much more commonly reported than cats, which may be a regional difference as cats are reported to be less popular in the north [26]. It may also be due to the nature of this sample being households with children, rather than all household types. However, in other childhood pet ownership data from a UK sample in the 1990s, cats were the most common pet type [19]. It is also noted that the age of the pet dogs reported was skewed towards younger age. This may be due to recall bias or guesswork in reporting of age of the dog by the children, older dogs in this population being more likely to be relinquished or abandoned, or most likely due to evidence that families with young children commonly do not acquire a dog or puppy until the child is older [19], meaning that by a child age 9–10 years the dog is still relatively young.

Girls were more likely to report owning pets than boys, for all pet types except rabbits. Previous research in the UK and Ireland suggests that females are more likely to own cats than males [15,17,18], and this relationship has also been suggested to apply to children in respect to cats, rabbits and rodents specifically but not other pets [19], or pets in general [27]. In contrast, others found no difference in pet ownership by gender [11,28]. Thus, there is good evidence that, during childhood, girls are more likely to own pets than boys, and it seems likely that this is a true phenomenon rather than girls just being more likely to tell us that they own a pet. However, there was no evidence that girls were more or less attached to their favourite pet than were the boys, which is similar to previous findings of no gender differences in frequency of play or care-giving related to pets owned by children [28], but contrasts with other studies, where females scored higher on pet attachment than did males [13,29]. These contrasting findings could reflect differences in the populations studied or the tools used to measure the relationship/attachment.

Ethnicity may act as an individual factor, although it frequently forms complex relationships with the religion, history and culture of specific ethnic groups; a simple measure of ‘ethnicity’ may not be fully representative of the beliefs and behaviour within that group. Due to the sample size we were limited in the analysis of ethnicity that could be performed. However, here we report that children of white ethnicity were more likely than non-white children to own dogs, rodents and ‘other’ pets. In data from the USA, whites have previously been observed to be more likely to own pets in adolescents aged 12–17 [11], in university students [13] and in the 21-64yrs age group [12]. We also found that white children were no more or less attached than non-white children to their favourite pet owned, disagreeing with previous findings which suggest higher white attachment [13,30] or rating of importance of the pet [11].

These differences may be due to limited sample sizes, combining results from ethnic minorities into a category of ‘non-white’ or, alternatively, may represent children and their families who have been domiciled in the UK for a prolonged period, even generations, and thus have incorporated 'western' influences. The use of different measures of relationship/attachment in each study may also contribute to differences in findings. It has been suggested that attachment scales may bias towards whites by considering western attitudes as a 'baseline,' rather than considering that western attitudes may in fact be more 'positive' and other ethnic attitudes 'baseline' [30]. More research into the potential cultural-bound phenomenon of the role of pets in the family is required [11], particularly in countries other than the USA. As some diseases or risk factors for disease are more common within specific ethnic groups, for example overweight and diabetes mellitus [31,32], pet ownership has the potential to counteract risk factors in these groups, via health promoting behaviours such as increased physical activity [33] and/or emotional support [1].

Previous research suggests that children with younger siblings have fewer pets than those with no younger siblings or singletons [27]; the presence of an older sibling increases the likelihood of ownership of dogs, rodents, birds and fish being reported [19]; or pet ownership does not vary with sibling status [11,28]. In our study, single children with no brothers and sisters were no more (or less) likely to own pets than those with younger brothers or sisters. We do, however, report novel and strong evidence that single children were more attached to their favourite pet, which concurs with the observation of Siegel (1995) that adolescents with no siblings in the household rated their pet as more important to them than those living with siblings. It has also been suggested previously, from parental report, that the youngest sibling plays more with a pet [28]. We also provide some evidence to suggest that children who were the youngest of their siblings were more attached to their favourite pet, although no more or less likely to report owning a pet in the first place.

Thus, research is contradictory as to whether presence of siblings (and their comparative ages) affects frequency of pet ownership, but single children and possibly youngest siblings may have stronger attachment to, and interact more with, their pets, than children with younger brothers and sisters. This is contrary to the findings of McConnell et al. (2011) who suggested that the support from pets provided (to adults) complements rather than competes with other human resources of support; however, they also noted that this required further investigation in people who are more socially isolated, as may be the case here of children with no brothers or sisters to play with.

There was good evidence to suggest that children who owned dogs lived in more deprived areas than those without dogs, strengthened by the dose–response relationship observed: the more deprived the area, the more dogs were owned. To our knowledge, this has not been previously reported, and may be unique to this particular area, although the findings are compatible with the general observation that dog ownership decreases as education level or social class of the owners increases [15,18,19,34]. Our data also suggested that ‘Pit Bull or cross’ or ‘Bull Breed’ dogs were more commonly found in more deprived areas, implying that ownership of these types of dogs has an inherent regional or cultural component linked to social deprivation. There was no evidence in our study that children who reported that their favourite dog was a Bull Breed were any more or less attached to the dog than those with a non-Bull-Breed dog. This fits with the observations of Maher and Pierpoint (2011) that so-called ‘status’ or ‘weapon’ dogs play a role of companionship, socialisation and protection in youth gangs in deprived inner city areas. There was also some suggestion from our data that non-white children were more likely to report owning a Pit Bull type (but not broader Bull Breeds) than white children. This may be a reflection of actual ownership of preferred dog types by different ethnic groups, or it could be due to non-whites being less inhibited in reporting that their dog is a Pit Bull, due to social and cultural reasons.

Two percent of all children sampled reported living with a ‘Pit Bull or cross’, and ten percent were living with similar ‘Bull Breed’ dogs including Staffordshire Bull Terriers, the most popular breed reported. Reported Pit Bull type dogs were surprisingly common, considering this is an illegal breed in the UK (Dangerous Dogs Act 1991), although not so surprising considering anecdotal knowledge of this region. Dog bites in this region have been reported in recent media, including fatalities in children, and are often attributed to Pit Bull and ‘status’ type breeds [35-37]. No data were collected on dog bites, and there was no specific mention of this information during conversation. Our data suggest that, if a considerable proportion of children are living with types of dogs that are often deemed to be ‘dangerous’ [23], the question can be raised as to why even more aggressive incidents are not reported.

Dogs were the pet that children most frequently reported owning, contacting outside their home, and were also the most common ‘favourite pet’ owned and scored the strongest attachment. This is likely to be due at least partly to real relationship differences between children and dogs compared to other pet types; Siegel (1995) also observed that fish owners felt that their pet was less important to them than dog or cat owners. However, it is also likely due to the nature of the questions asked in the CENSHARE Pet Attachment Scale, it was impossible to score highly when answering questions about a pet fish, as they are very unlikely to sleep on the bed or require daily grooming, and this was something that a number of children were observed to be frustrated by when completing this part of the questionnaire. This is an issue that should be addressed in future studies of this nature.

## Conclusions

Data on childhood pet ownership is scarce but essential if we are to understand the impact of the common phenomenon of growing up with pets, in terms of both benefits and risks. This study provides evidence that, at least in this region, gender, ethnicity and socioeconomic status are associated with pet ownership, and sibling status is associated with level of attachment to the pet. There is also evidence that ownership of dogs, particularly bull breeds, is associated with increased deprivation. This increases our understanding of who is likely to own which pet types, and the nature of relationships that children can have with their pets.

## Competing interests

We declare that authors: (1) CW, SD, RG, PB, AG, and KC have institutional support via a grant from Mars Petcare for the submitted work; the previous position of CW was funded from this grant; (2) the position of AG at the institution is funded by Royal Canin, a subsidiary of Mars Petcare and he has also received other research grants from Mars Petcare; (3) SMcC is an employee (Research Manager) of WALTHAM, a division of Mars Inc; (4) LB and GS have no financial or personal relationships with other people or organisations that could inappropriately or influence or bias the content of the paper.

## Authors’ contributions

CW conceived and designed the study, collected the data, performed the data analysis and drafted the paper. LB and GS provided access to data collection and advised on study design and data analysis. SD, PB, AG, RG, and KC were involved in conception of the study, study design and interpretation of findings. AG and KC also assisted with data collection and SD was also principal investigator. SMcC assisted in study design and interpretation of findings. All authors read and approved the final manuscript.
